# The long-term collateral consequences of juvenile justice involvement for females

**DOI:** 10.3389/fpsyg.2023.1321355

**Published:** 2024-01-08

**Authors:** Maria L. Schweer-Collins, Carly B. Dierkhising, Leslie D. Leve

**Affiliations:** ^1^Prevention Science Institute, University of Oregon, Eugene, OR, United States; ^2^School of Criminal Justice and Criminalistics, California State University, Los Angeles, CA, United States; ^3^Department of Counseling Psychology and Human Services, College of Education, University of Oregon, Eugene, OR, United States

**Keywords:** developmental psychopathology, trauma, offending, incarceration, juvenile justice, longitudinal research

## Abstract

**Introduction:**

Females are the fastest growing justice involved population in the United States, yet there is relatively little empirical research on the collateral consequences of juvenile justice involvement specifically for females. A growing body of empirical research underscores linkages between juvenile justice involvement and negative health and psychosocial outcomes, both in the short and long term.

**Method:**

The current study describes the long-term collateral consequences of juvenile justice involvement for females previously involved in the juvenile justice system, drawing from a longitudinal dataset of 166 women who were initially recruited in adolescence due to chronic and severe justice system involvement. Participants were 15 years-old on average at study enrollment and 35 years-old on average at the current assessment. This paper describes the adolescent and adult experiences of the sample, therefore depicting the developmental trajectories of risk and protective factors for females involved with juvenile justice.

**Results:**

As adults, 73% of the sample experienced arrest and 36% experienced incarceration. High rates of mental and physical health problems were reported, including that 50% of the sample met diagnostic criteria for posttraumatic stress disorder. Over 400 children were born to the sample, with high rates of documented intergenerational child welfare involvement.

**Discussion:**

Study findings are discussed in the context of best practices for supporting adolescent girls involved with the juvenile justice system.

## Introduction

Females are the fastest growing justice involved population in the United States, with nearly 1 million females under the supervision of the criminal justice system (Bureau of Justice Statistics, 2023). In 2022, 14% of incarcerated persons in jails were women and from 2021 to 2022, the female population grew at a faster rate (9%) than the male population (3%) (Bureau of Justice Statistics, 2023). The overall rate of juvenile arrests for both females and males has declined in the past decade, yet the decline has been greater for male youth (Puzzanchera, 2021). Although researchers and policymakers increasingly recognize the importance of understanding the unique risk and protective factors related to female’s involvement in the justice system (Zahn et al., 2010; Lynch et al., 2012; Kerig, 2018) and exploring gender-responsive approaches within the context of juvenile justice (Kerig and Schindler, 2013), there is relatively little empirical research on the collateral consequences of juvenile justice involvement specifically for females.

A growing body of empirical research underscores the detrimental effects of juvenile justice involvement, both in the short and long term. This literature reveals that youth incarceration not only fails to effectively reduce crime rates but also leads to a myriad of adverse collateral consequences (Holman and Ziedenberg, 2013; McCarthy et al., 2016; Mendel, 2022b). Among these collateral consequences is the association between juvenile incarceration and the exacerbation of mental health problems. For example, high rates of posttraumatic stress and depression symptoms have been observed following juvenile incarceration and are associated with the traumatic experiences youth report while in secure confinement (Dierkhising et al., 2014). Collateral consequences of juvenile justice involvement are far reaching and include barriers to housing, employment, education, and public benefits (Dierkhising et al., 2023). Juvenile incarceration has been causally linked to lower academic attainment and adult criminal justice involvement (Aizer and Doyle, 2015). Adolescents with juvenile justice histories are ultimately burdened with less social and economic capital as they enter young adulthood.

The literature on collateral consequences of juvenile justice involvement has not specifically focused on females. However, much is known about the extensive histories of trauma and victimization, particularly experiences of sexual violence (Johansson and Kempf-Leonard, 2009; Kerig and Becker, 2010), that females involved in the juvenile justice system have experienced. Females often come to the system and/or are confined for offenses related to their experiences of violence and victimization (e.g., running away, truancy, housing instability, commercial sexual exploitation); for example, in 2021 females were more likely to be detained for status offenses (24%) compared to males (19%) (Puzzanchera et al., 2023). In addition, females involved in the juvenile justice system are two times more likely to report a history of sexual abuse (16% vs. 32%) and four times more likely to report a history of sexual assault/rape (9% vs. 39%) compared to males (Dierkhising et al., 2013). These findings have led to the identification of a sexual violence to prison pipeline that is often the trajectory for females (Saar et al., 2015). Females, particularly those in secure confinement, are also at increased risk for additional trauma exposure once system involved (Saar et al., 2015). For example, youth with histories of sexual abuse or assault are at greater risk for sexual abuse or assault during incarceration (Dierkhising et al., 2014). In addition, there is growing recognition that females are more likely to be held in secure confinement, often for lower-level offenses, not for public safety, but because they are seen as vulnerable (Beck et al., 2010). This approach is deeply rooted in gender biases (Spivak et al., 2014), and highlights the need for a deeper understanding of the collateral consequences of justice involvement among females in order to identify more appropriate alternatives to detention or prevention and intervention options for this population.

**Figure 1:**
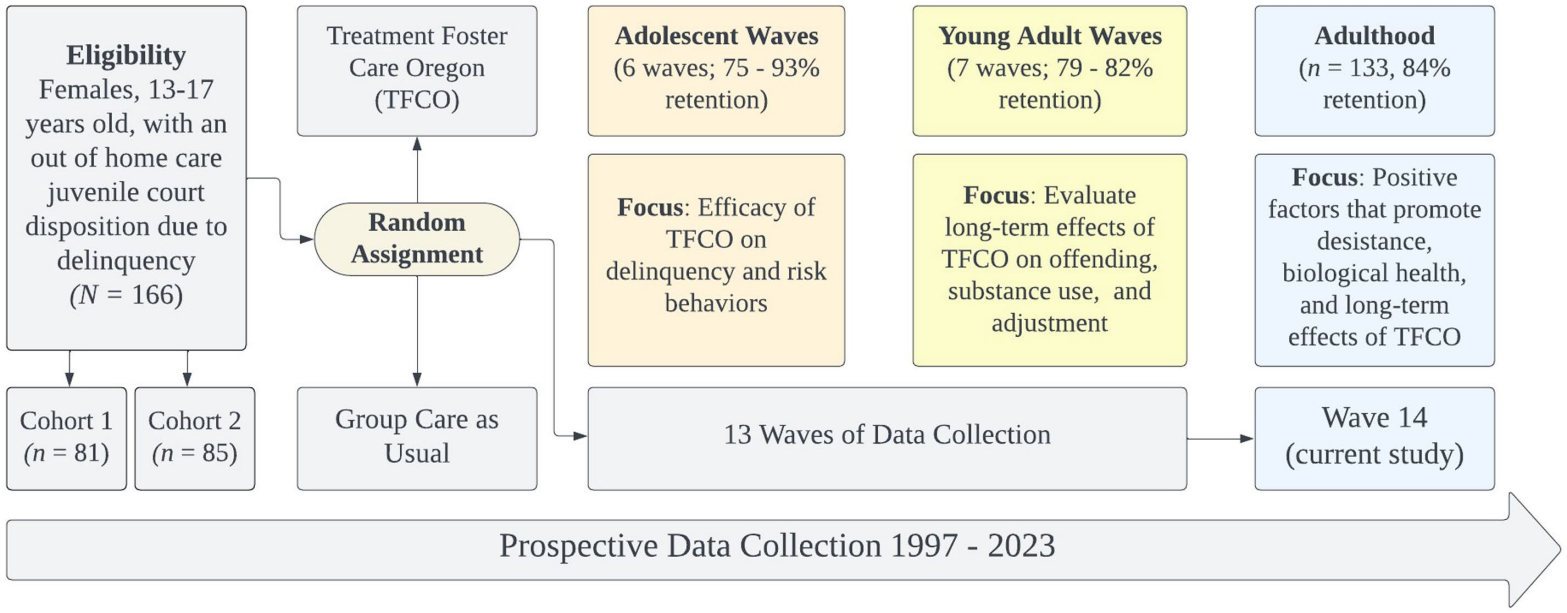
Description of the turning points for women study.

In this paper, we describe the long-term collateral consequences for females involved in juvenile justice. To do so, we draw from a rich, longitudinal dataset of 166 women who were initially recruited in adolescence due to serious and chronic involvement with juvenile justice^1^ (Chamberlain et al., 2007). The sample has been followed for the past 26 years with 14 different assessment points (see Figure 1). The current assessment wave includes the Turning Points for Women Study (TPWS), which is unique because prospective, longitudinal studies in criminology rarely focus on women and those that exist, often follow community samples (e.g., The Pittsburgh Girls Study [PGS]; Keenan et al., 2010). A small number of high-quality prospective, longitudinal studies have examined patterns of offending from adolescence into adulthood and have included subsamples of females (e.g., Pathways to Desistance, 16% female; Mulvey, 2004; The Northwestern Juvenile Project, 36% female; Teplin et al., 2013). TPWS is an important complement to the existing literature and well equipped to understand the collateral consequences of female justice involvement because it: (1) involves a robust assessment battery across substance use, psychiatric disorders, trauma history, official records of offending and types of offenses; (2) includes trauma history information and assessment of multi-system involvement (e.g., child welfare, juvenile justice, criminal justice); (3) follows a sample that not only had serious and chronic delinquency during adolescence but was court-mandated to out of home care, (4) documents biological indicators of stress and health. Therefore, the objective of the current paper is to provide rich, descriptive data on the adolescent and early adulthood experiences of this unique sample of women to characterize the psychosocial, health, relational, and systems involvement consequences they have sustained.^2^

## Methods

### Participants

The participants in this study are 166 females who were followed for the past 18–26 years, beginning in either 1997 (Cohort 1) or 2003 (Cohort 2). Participants were 13–17 years (*M* = 15.31, *SD* = 1.17) at study enrollment. At the final wave of data collection, participants were 29–42 years old, (*M* = 35.0, *SD* = 2.93). The self-reported racial and ethnic breakdown of the full sample was: 68.1% non-Hispanic White, 1.8% African American, 11.4% Hispanic, 0.6% Native American, 0.6% Asian, 16.9% multi-ethnic/racial heritage. At the most recent wave of data collection, the sample maintained similar self-reported racial and ethnic proportions: 68.9% non-Hispanic White, 1.6% African American, 11.6% Hispanic, 0.1% Native American, 0.1% Asian, 17.8% multi-ethnic/racial heritage. At the final assessment wave, participants self-reported gender identity and all participants who completed the assessment self-identified as women (*n* = 129). The participants reported their sexuality as heterosexual (66%), lesbian (4%), bisexual (26%), pansexual (2%), and 2% declined to answer.

### Measures

#### Youth variables

##### Arrests and offenses

Age at first arrest and frequency counts of offenses, arrests, were all measured prior to age eighteen and are based on official records. Offenses include all types (i.e., status, misdemeanors, felonies) and are nested in arrests as youth can be charged with multiple offenses at one time of arrest (Chamberlain et al., 2007; Leve et al., 2022). Days in locked settings was based on participant self-report. Participants were asked at each wave of the study where they were and total days in locked settings at any point prior to the participant’s eighteenth birthday was computed (see Supplementary File A for details).

##### Foster care history

Number of foster care placements prior to age eighteen includes both official child welfare records and case worker report and, when child welfare records were not available, parent and self-report of all placement changes (unpublished measure, Oregon Social Learning Center; see Supplementary File A for details). It includes all out of home care experiences that were not related to delinquency court dispositions.

##### Adverse childhood experiences

A 10-item adverse childhood experiences (ACE) revised composite score was created using two items from youth self-report; seven items from caseworker report; and one coded item from official maltreatment records. All items were measured at baseline. Items were selected based on the original ACE measure (Felitti et al., 1998). All items were coded 0 (*no*) and 1 (*yes*) for the presence or absence of each risk factor. Items were summed to create a risk index ranging from 0 to 10, with higher scores reflecting greater ACEs. See Supplementary File B for the wording and scoring of items.

##### Mental health

Mental health diagnoses were measured through self-report at baseline using the Brief Symptom Inventory (BSI; Derogatis and Melisaratos, 1983) and included the proportion of those who met symptom threshold criteria for clinical levels of depression, anxiety, or both. If participants scored above 91 on T-scores for the depression and anxiety subscales, they were identified as having clinically elevated depression and anxiety symptoms, respectively. Cronbach’s alphas for the depression and anxiety subscales were 0.80 and 0.88, respectively.

##### Substance use

Participants were asked three questions at baseline regarding whether in the past year they had used alcohol, cannabis, and illicit drugs such as uppers, downers, or acid (i.e., drugs other than tobacco, alcohol, or cannabis). Responses were provided based on frequency of use as either, 1 = never, 2 = once or twice, 3 = occasionally, 4 = 1–6 times per week, 5 = 1 or more times per day. Thus, substance use variables were continuous variables, with lower scores indicating no alcohol, cannabis, or illicit substance use and higher scores indicating more severe substance use.

#### Adult variables

##### Adult criminal legal system involvement

Adult criminal legal system data were collected from official arrest records during each of the young adult and adult waves of the study. Records were collected for 100% of participants in both the young adult and adult phases of the study. The original study gathered residential history information (self-reported) from all participants, and then attempted to collect criminal record data from every county in which the participant lived for the duration of the study. We produced a complete record of each participant’s official criminal records by compiling the offenses at each assessment (Leve et al., 2022). We then removed any duplicate offenses that occurred because of the record compilation process. We computed the age of first adult arrest using participant date of birth and the date of the first arrest which occurred on or after the 18th birthday. If a participant self-reported an arrest, but no official arrest records data were found, we conservatively used the participant’s self-report of arrest.

##### Self-reported health

Self-reported health was measured using a modified 12-item version of the RAND 36-item Short Form Survey (Ware and Sherbourne, 1992). For self-reported general health, participants responded to five questions (e.g., “In general, would you say your health is:”) and rated their health from 1 – “excellent” to 5 – “poor.” Items were then reverse scored, with ratings of poor (5) being given a score of “0” and ratings of excellent (1) being given a score of “100,” and averaged. To assess chronic health conditions, participants reported yes or no as to whether they had ever been diagnosed or treated with 19 chronic health conditions. We provided data on the top five reported chronic health conditions and the proportion of participants who endorsed those conditions.

##### Mental health

To assess whether participants met clinical diagnostic thresholds for depression and anxiety, we used the Brief Symptom Inventory (BSI; Derogatis and Melisaratos, 1983). As with the youth measure, if participants scored above 91 on T-scores for the depression and anxiety subscales, they were identified as having clinically elevated depression and anxiety symptoms. Cronbach’s alphas for the anxiety and depression subscales were 0.79 and 0.85, respectively. Participants also self-reported as to whether they had ever been diagnosed or treated for depression or anxiety, for example, “Are you now or have you ever been treated for or diagnosed with depression [anxiety]?” To determine if participants met current diagnostic criteria for posttraumatic stress disorder (PTSD), the International Trauma Questionnaire was used (ITQ; Cloitre et al., 2018) The ITQ has nine symptom indicators of PTSD and participants are documented to have diagnosable PTSD if they endorse at least one symptom from symptoms clusters including (1) re-experiencing in the here and now, (2) avoidance, and (3) sense of current threat. Participants must also endorse functional impairment due to these symptoms by endorsing one of three items related to functional impairment.

##### Substance use

Alcohol use was measured in this study through measures of number of drinking days in the past 6 months (“How many days did you drink beer, wine or hard liquor in the last six months?”) and number of drinks in a drinking day (“Thinking about all types of alcohol, like beer, wine, or hard liquor, how many drinks have you usually had at one time in the past 6 months?”). Cannabis use, opioid use, and other illicit drug use was also reported on for the past 6 months. In the illicit drug use category, participants were asked about use of the following illicit drugs: inhalants, hallucinogens, club drugs, prescription drugs for non-prescription use; stimulants, and downers. If participants indicated they had used any of the listed illicit drugs, opiates, or cannabis in the past six months, they were indicated as having that specific drug use. Participants self-reported whether they had ever been diagnosed or treated for a substance use disorder (“Are you now or have you ever been treated for or diagnosed with alcohol or drug abuse?”). All questions for substance use were drawn from an unpublished measure (Rhoades et al., 2014) but are aligned with typical retrospective reports of substance use similar to the widely used Timeline Follow-Back procedure (Sobell and Sobell, 1992).

##### Educational and socioeconomic outcomes

Participants were asked one question about educational attainment (“What is the highest level of education you have completed?”). Participants were asked about their household income (“What is your annual (gross) household income?”) and how many people the household income supported (“How many people does that income support?”). To assess the poverty level of the sample, we computed poverty threshold by dividing household income by the federal poverty level in relation to the number of individuals in the household. Next, we transferred this number into a percent poverty level by multiplying the number by 100. To assess subjective socioeconomic status (SES), we used the MacArthur Scale of Subjective Social Status – Adult Version (Adler et al., 1994). Participants were shown a ladder and asked where they placed themselves on the ladder in relation to other people in terms of money, education, and jobs.

#### Family and relational outcomes

##### Pregnancy and childbirth

Data on pregnancy and childbirth were obtained prospectively at each wave of the study. During adolescence, participants and caregivers were separately interviewed regarding the youth’s pregnancies and pregnancy outcomes (i.e., miscarriage, induced abortion, live birth, stillbirth) that had occurred at each assessment wave. Caregiver report was used only when adolescent report was unavailable (Kerr et al., 2009). In adulthood, participants self-reported this information. The presence or absence of a pregnancy, stillbirth, induced abortion, miscarriage, and live birth was coded as 1 = yes and 0 = no to indicate presence or absence of a pregnancy or pregnancy outcome that occurred at any wave of the study (Cioffi et al., 2022). The reported date of the first pregnancy and childbirth was used along with participant date of birth to compute the first pregnancy and childbirth variables, only for participants with histories of pregnancy and childbirth.

##### Child welfare contact

To determine the number of children placed into child welfare custody, official child welfare records were obtained in the young adult assessment (Leve et al., 2015). Participants also self-reported custody status at each wave of the study, and in cases where the child was not in the custody of the study participant, the custodial caregiver data was collected, including whether custody was held by “the state, SCF, child welfare, or DHS.” In the final assessment, if official child welfare records were not available for a newly born child, self-report of child custody status was used, but in all other cases, child welfare involvement was indexed through official records.

##### Intimate partner relationships

Current relationship status was assessed by three questions asking if participants were currently in a relationship, cohabitating with their current partner, and currently married. Intimate partner violence was assessed through the participant’s report on the Revised Conflict Tactics – Short Form (CTS2S; Straus and Douglas, 2004). The CTS2S is a 20-item instrument that assesses negotiation, physical and sexual assault, and psychological aggression in couple relationships. Participants reported on the frequency of each behavior, including their own behaviors, on a scale from 1 (“once in the past year”) to 8 (“this has never happened”). In this study, participants were categorized into having experiences of intimate partner violence if they reported being a victim, perpetrator, or both a victim and perpetrator on the mutuality scores of psychological aggression, injury, or physical or sexual assault in their current relationship. The CTS2S authors note that internal consistency is not appropriate for the measure and therefore it is not presented here.

Couple adjustment was assessed through the Dyadic Adjustment Scale (DAS; Spanier, 1976). The DAS is a 32-item questionnaire that comprises four subscales (Consensus, Satisfaction, Cohesion, and Affectional Expression). The full-scale score (range = 0 to 151) was used in the present study, and higher scores reflect better dyadic adjustment and scores above 114.8 reflecting better adjustment and a happier couple. Conversely, participants in this study were indicated to be in a distressed relationship if they were at or below the cut-score of 98. Cronbach’s alpha for the total scale was 0.94.

## Results

### Adolescent outcomes

Participants became involved with the juvenile justice system, on average, at age 12.69 (*range* 6.52–16.47). The average number of arrests prior to age 18 was 15.5 (*SD* = 10.3), with the average number of times assigned to a locked setting prior to age 18 being 4.28 (*SD* = 3.15), and the average number of days spent in a locked setting prior to age 18 being 193 (*SD* = 263). All participants were adjudicated delinquent.

Many participants also were involved with the child welfare system as children (88.6%), and of those, many experienced out of home care with the average number of foster care placements being 3.48 (*SD* = 4.54). Overall, ACEs were high in this sample (*M* = 5.71, *SD* = 2.17), with interpersonal violence or physical abuse in the immediate family (but not directed at participant) being the most commonly experienced ACE (75.3%), followed by having a parent incarcerated (71.7%), and child sexual abuse (62%). Four other ACEs were experienced by close to or just above 50% of the sample (physical abuse of the participant, emotional neglect, parental substance use, parental divorce).

Participants also experienced mental health challenges, including clinically elevated anxiety symptoms (18.7%) and depression symptoms (12%). On average, the participants reported occasional past 12-month use of alcohol (*M* = 3.07, *SD* = 1.35) and cannabis (*M* = 3.12, *SD* = 1.50) on a scale from 1 = never used to 5 = 1 or more times per day). Participants also endorsed, on average, using illicit substances once or twice in the past 12 months (*M* = 2.49; *SD* = 1.41). See Table 1 for complete reporting of adolescent outcomes.

**Table 1:** Sample characteristics from adolescence, under 18 years of age.

Variables	*M* (*SD*) or *n* (% of total sample)	TFCO intervention effect found
**Juvenile justice contact**		
Age (years) at first arrest	12.69 (1.70)	
Range of ages at first arrest	6.52–16.47	
Number of offenses <18	15.5 (10.3)	*
Range of number of offenses	1–63	
Number of times in locked setting	4.28 (3.15)	
Range of number of times in locked setting	0–16	
Days in locked setting <18	192.95 (263)	*
Range of days in locked setting <18	0–1,644	
**Child welfare contact**		
Number of foster placements <18	3.48 (4.54)	*
Range	0–23	
**Adverse childhood experiences**		
ACEs Total	5.71 (2.17)	
ACE Total Range	0–10	
1. Emotional abuse	61 (36.7%)	
2. Physical abuse	107 (54.5%)	
3. Child welfare documentation of sexual abuse	103 (62.0%)	
4. Emotional neglect	90 (54.2%)	
5. Neglect: failure to provide	22 (13.2%)	
6. Parental divorce	97 (58.4%)	
7. Interpersonal violence or physical abuse in immediate family	125 (75.3%)	
8. Biological or step-parent has history of drug or alcohol abuse	80 (48.2%)	
9. Biological or step-parent was hospitalized for mental illness	47 (28.3%)	
10. Biological or step-parent was convicted of a crime	119 (71.7%)	
**Mental health diagnosis**		
Depression	20 (12.0%)	
Anxiety	31 (18.7%)	
**Substance use outcomes** ^**a**^		
Alcohol use level past 12 months	3.06 (1.35)	
Cannabis use level past 12 months	3.12 (1.50)	
Illicit substance use level past 12 months	2.49 (1.41)	

a Substance use 1 = never, 2 = once or twice, 3 = occasionally, 4 = 1–6 times per week, 5 = 1 or more times per day. *Note:* ^*^indicates that a significant intervention effect has been found on this outcome variable at *p* < .05.

### Adult outcomes

The majority of the sample had involvement with the adult legal system (*n* = 121, 73%), with the average age at first adult arrest being 19.88 (*SD* = 2.14). The average number of adult offenses was 6.72 with wide variability (*range* = 0–68). Of the sample, 35.5% had been incarcerated as an adult. Age at last arrest was, on average, 22.80 (range = 12–37). At the final assessment wave, two participants (1.2%) were currently incarcerated.

On average, participants reported their general health was 46.93 (*SD* = 21.70), slightly under a value of 50, which is considered normative general health. Participants endorsed a variety of chronic health conditions, with the two most common being asthma (*n* = 58, 45%) and anemia (*n* = 50, 38.8%). At the time of this most recent assessment wave, eight of the original participants were known to be deceased (4.8%). Posttraumatic stress disorder was the most common diagnosis, with 65 (50%) participants meeting diagnostic criteria for PTSD, and 15 (11.6%) and 17 (13.1%) meeting criteria for depression and anxiety, respectively. Alcohol was the most commonly endorsed substance, with 72 (55.8%) of participants reporting using alcohol in the past six months. For those who consumed alcohol, the average number of drinking days in the past six months was 38.1 (*SD* 51.72). Rates of cannabis use were also high, with 59 (45.7%) participants reporting past 6-month use of cannabis; 54 (41.9%) reported past 6-month use of illicit drugs, and 21 (16.3%) reported past 6-month use of opioids.

The most common level of educational attainment was a high school diploma or GED (34%), and 60 participants reported being currently employed (46.5%). The average annual income of the sample was $33,771.46 supporting, on average, 3.11 individuals (*SD* = 1.85). The sample was, on average, 134% (*SD* = 110%) above the 2023 U.S. federal poverty thresholds when accounting for household size (Office of the Assistant Secretary for Planning and Evaluation [ASPE], 2023).

For those who had ever experienced a pregnancy, the average age at first pregnancy was 16.90 (*SD* = 3.09) and average age at first childbirth was 19.82 (*SD* = 2.55). Of the 408 children born to participants in this sample, 127 (31.3%) had histories of being in custody of child welfare, and 64 participants (38.6%) had official documentation of involvement with child welfare as a parent.

The majority of participants were partnered (*n* = 96, 74.4%), primarily cohabitating with their partner (51.9%) or married (19.4%). Of those participants who were in current relationships, 76 (58.9%) reported an experience of interpersonal violence in the current relationship. Conversely, 48 (37.2%) participants reported dyadic adjustment indicative of a satisfied and happy couple relationship. See Table 2 for complete reporting of adult outcomes.

**Table 2:** Sample characteristics from adult waves, 18 years of age and older.

Variables	*M* (*SD*) or *n* (% sample^a^)	Range^b^	Wave
**Adult criminal legal system involvement**			
Percent of sample with adult arrest	121 (73%)	–	AW
Age (years) at first adult arrest	19.88 (2.14)	18–27	AW
Age at last adult arrest	22.80 (6.34)	12–37	TP
Number of offenses >18	6.72 (11.41)	0–68	AW
Adult Incarceration (jail/prison sentence)	59 (35.5%)	–	AW
Length of jail/prison sentence (days)	230.36 (823.97)	2–5,610	AW
Currently incarcerated	2 (1.2%)	–	TP
**Health outcomes**			
***Physical health***			
Self-reported health	46.93 (21.70)	0–100	TP
Top 5 chronic health conditions			TP
Asthma	58 (45.0%)	–	
Anemia	50 (38.8%)	–	
Obesity	27 (20.9%)	–	
High blood pressure	23 (17.8%)	–	
Musculoskeletal condition (e.g., arthritis)	20 (15.5%)	–	
Deceased	8 (4.8%)	–	AW
***Mental health***			
Current clinical depression symptoms	15 (11.6%)		TP
Current clinical anxiety symptoms	17 (13.1%)		TP
Current posttraumatic stress disorder diagnosis	65 (50.1%)		TP
Lifetime history of treatment and/or diagnosis of depression	83 (64.3%)		TP
Lifetime history of treatment and/or diagnosis of anxiety	81 (62.7%)		TP
***Substance use***			
Alcohol use (ever used in past 6 months)	72 (55.8%)	–	TP
Alcohol (number of drinking days in past 6 months)	38.1 (51.72)	0–180	TP
Alcohol (number of drinks in drinking day)	3.5 (3.58)	0–24	TP
Cannabis use (ever used in past 6 months)	59 (45.7%)	–	TP
Illicit drug use (ever used in past 6 months)	54 (41.9%)	–	TP
Opioid use (ever used in past 6 months)	21 (16.3%)	–	TP
Self-reported substance use dependence diagnosis	71 (42.8%)	–	TP
**Educational and socioeconomic outcomes**			
Educational attainment			TP
Below High School	25 (19.4%)	–	
High school diploma or GED	44 (34%)	–	
Some college, no degree	38 (29.5%)	–	
A.A. or Vocational degree	12 (9.3%)	–	
4-year college degree	6 (4.7%)		
Proportion employed	60 (46.5%)	–	TP
***Socioeconomic outcomes***			
Household income	$33771.46 ($29258.35)	$0–180,000	TP
Poverty level based on federal guidelines	134.40% (110.85%)	−39 to 542.6%	TP
Number of people supported by income	3.11 (1.85)	1–9	TP
Subjective social status	3.66 (1.69)	1–8	TP
**Family and relationship outcomes**			
***Children/pregnancies***			
No. of children born in sample	408	–	AW
Pregnancy losses in sample (miscarriage, abortion, stillbirth)	309	–	AW
Age at first pregnancy	16.90 (3.09)	7–30	AW
Age at first childbirth	19.82 (2.55)	8–29	AW
***Child welfare contact***			
Participant with official report of child welfare involvement (as parent) ever	64 (38.6%)	–	AW
Total no. of children out of custody related to child welfare involvement ever	127 (31.1%)	–	AW
***Intimate partner relationships – current relationship***			
Relationship status			TP
Single	33 (25.6%)	–	
In couple relationship – not cohabitating	4 (3.1%)	–	
In couple relationship – cohabitating	67 (51.9%)	–	
Married	25 (19.4%)	–	
Experience of interpersonal violence	76 (58.9%)	–	TP
Couple dyadic adjustment			TP
Satisfied and happy couple relationship	48 (37.2%)	–	
Distressed couple relationship	24 (18.6%)	–	
Neither distressed nor satisfied/happy couple relationship	24 (18.6%)	–	

AW, data drawn from all available waves with a sample size of *n* = 166; YA, data drawn from young adult wave with a sample size of *n* = 154; TP, data drawn from most recent wave called Turning Points with a sample size of *n* = 129; No., Number.

a Percentages may not equal 100% in each category due to missing data, participant not eligible to respond, or declined responses.

b Ranges, when applicable, are those observed in the sample.

## Discussion

The current study showed that among this group of women who had experienced high levels of arrests, out of home placements, and incarceration as juveniles, many continued to have involvement in the justice system in adulthood. Nearly three-fourths of the women were involved with the adult criminal legal system, yet, the last arrest was, on average, at age 22 years indicating a pattern consistent with the age-crime curve: high offending in adolescence with a sharp decline in offending into adulthood (Farrington, 1986; Moffitt, 1993; Piquero et al., 2003). The variability in the age range of 12–37 years at last arrest also indicates both desistance and persistence within the sample.

Women in the sample experienced significant early adversity, with an average of nearly six ACEs. This is striking as data show the majority of U.S. adults have experienced three or fewer ACEs (Swedo et al., 2023). Given the high ACEs in this sample and the known link between ACEs and health problems (Felitti et al., 1998), it is not surprising to also see high rates of mental and physical health concerns in adulthood. Notably, nearly two-thirds of the sample had experienced child sexual abuse, which is likely an underestimate given this was based on caseworker report or official cases in the child welfare system. This finding provides empirical support to the emerging theory on the sexual violence to prison pipeline among females (Saar et al., 2015). Overall, the sample experienced significant trauma in childhood and adolescence as documented by their ACEs, involvement in the child welfare system, and high prevalence of mental health concerns in their initial assessment. Ongoing system involvement was also prevalent and likely contributed to ongoing stress and trauma in the lives of the females as they entered adulthood. Indeed, nearly two-thirds of the sample reported a lifetime history of treatment and/or a diagnosis of anxiety and/or depression, and half of women (50%) met criteria for a diagnosis of PTSD at the most recent assessment in adulthood. This statistic reflects their symptom expression at the time of the assessment. In comparison, lifetime PTSD rates in the general population are estimated at 8% among women (Lehavot et al., 2018). Thus, the prevalence in this sample is 6.25 times the prevalence rate among the general population of women and is also underestimated given it does not include lifetime prevalence.

The physical health outcomes shown in this study as participants approached middle adulthood corroborate evidence of the known physiological burden of chronic psychosocial and systems stress on the body (McEwen and Stellar, 1993; Guidi et al., 2021). Participants self-reported below average levels of health, rating their general health with the SF-36 at 46 on a scale of 0–100, where 50 is considered average health. Conversely, a normative score on general health for women in the U.S. is 70.6, corresponding to “good” to “very good” general health (Obidoa et al., 2010). Females in the study also endorsed high rates of chronic health conditions. For example, more than one-third of the sample reported experiencing asthma and anemia, and 15%–20% of the sample reported experiencing obesity, high blood pressure, and musculoskeletal conditions such as arthritis. These conditions may be indicative of greater allostatic load (Guidi et al., 2021) and many of these chronic conditions place individuals at risk for cardiometabolic morbidity and subsequently, premature or increased mortality (Reilly and Kelly, 2011; Xu et al., 2018; World Health Organization [WHO], 2021), which was also observed in this sample as eight participants (nearly 5%) were deceased by the last wave of this study.

Nearly nine out of ten females were dual system youth, youth who experienced both juvenile justice and child welfare system involvement. Prevalence rates of dual system involvement are generally 50%–70% (Herz et al., 2019, 2021). However, dual system youth are more likely to be female and tend to experience more out of home care while in either child welfare or juvenile justice compared to the general juvenile justice population (Herz et al., 2019). Given that the current sample was recruited based on their gender and disposition for out of home care it is not surprising the prevalence of dual system involvement is higher in the current sample.

Perhaps most striking are the outcomes related to pregnancy and parenting. The total number of pregnancies in the sample was 717, with 309 (43%) resulting in loss (i.e., miscarriage, abortion, stillbirth). The majority of first pregnancy and childbirth experiences were in adolescence at, on average, 16 and 19 years old, respectively. Of the 408 children born to the full sample of 166 females, nearly two in five children were referred to the child welfare system and nearly one-third experienced out of home care (e.g., foster care). These findings are aligned with the literature on pregnant and parenting youth with histories in foster care (Eastman et al., 2019), which reveal that one in three females in foster care give birth by age 21 (Putnam-Hornstein et al., 2016). The high levels of involvement with child welfare that participants experienced in their childhood and again as parents also corroborate research documenting intergenerational involvement in the child welfare system among youth in the juvenile justice system (Putnam-Hornstein et al., 2015). Research also reveals that risk for intergenerational involvement in child welfare is nuanced, with mothers with mental health problems (Hammond et al., 2017), those experiencing housing instability while in out of home care, and those living in congregate care at the time of birth (Eastman and Putnam-Hornstein, 2019) at greatest risk for intergenerational involvement in child welfare. Future research should seek to understand the protective factors that buffer risk for intergenerational involvement in child welfare, particularly in females with multiple intersecting risk factors. Such findings will be central to developing tailored supports for pregnant or parenting females involved with the juvenile justice system.

The majority of the sample reported being in a relationship (74.4%). Although approximately one-third of participants reported positive dyadic adjustment, reaching levels indicative of a satisfied and happy couple relationship (Spanier, 1976), 59% of participants also reported experiencing some form of intimate partner violence (IPV) in their current relationship. This included being a victim of psychological, sexual, or physical abuse or mutually engaging in these behaviors in the relationship with their significant other. These findings are consistent with empirical studies showing that IPV is a correlate and predictor of crime, arrest, and incarceration for women (Brennan et al., 2012; DeHart et al., 2014; Gottlieb and Mahabir, 2021), yet they highlight the need for the legal system to consider IPV when considering the best ways to support women who come into contact with the system. Further, prior research has documented that women may show desistance through supportive relationships (Rodermond et al., 2016), demonstrating the need for examining nuanced measures of relational quality, including positive aspects of the couple relationship, as predictors of legal systems involvement.

### Limitations

This study described the collateral consequences of females who, from early adolescence, experienced extensive involvement in the juvenile justice system. Although data were collected prospectively, we note that this paper does not describe the causal outcomes of juvenile justice involvement. This is an important limitation as it is well documented that youth who enter the juvenile justice system are also more likely to have multiple risk factors prior to entry that may be linked with the psychosocial, relational, and health consequences outlined in this paper. In addition, the sample had severe and chronic juvenile justice system involvement, and thus the current results may not extend to women with lighter involvement in the system (e.g., no out of home care), to males, or to women from other geographic regions or racial-ethnic backgrounds. Additionally, data on participant experiences of interpersonal violence was collected through self-report and therefore, participants may have underreported or minimized their experiences.

### Future directions

The current study documents the many potential risks and outcomes that may be associated with severe and persistent juvenile justice system involvement for females. In addition to the consequences of justice system involvement, research documents that females enter the juvenile justice system with significant trauma histories (Kerig and Becker, 2010; Anderson and Walerych, 2019) and are frequently incarcerated for reasons unrelated to public safety. There are now initiatives to *end* the juvenile incarceration of females. Hawaii, for example, had zero females incarcerated in their youth facility as of 2022 (Healy, 2022) and in California, the Vera Institute of Justice, is working at the County level to end the incarceration of females and gender expansive youth (Vera Institute, 2023). A primary goal of these initiatives is to serve youth in their community and/or divert youth from the system entirely. Community-based approaches to youth arrests and increases in diversion options have become best practice across the nation in order to reduce confinement of youth and the collateral consequences of juvenile justice involvement (Mendel, 2022a, 2023). Findings from the current study support these efforts and future research should evaluate how alternatives to incarceration for females differentially relate to the long-term collateral consequences of juvenile justice involvement for females.

Future research efforts with the TPWS will disentangle the effects of juvenile justice and adult criminal legal system involvement, while considering other salient life events such as trauma, relationships, parenting, and involvement in multiple public systems, that may function as turning points to explain the heterogeneity in outcomes among the women. As suggested in this paper and in line with a developmental psychopathology perspective and emerging work in positive criminology (Ronel and Elisha, 2011; Ronel and Segev, 2014; Kewley, 2017), it is imperative that future research measure and explore positive, protective factors across intra and interpersonal, and systemic levels to advance a nuanced understanding of how an individual harnesses internal resiliency and/or benefits from social supports as they desist from crime.

## Data availability statement

The datasets presented in this article are not readily available because data generated with NIJ grant 2020-JX-FX-0003 will be made publicly available through the National Archive of Criminal Justice Data at the end of the funding period (December 31, 2023) and in accordance with funder requirements. Requests to access the datasets should be directed to https://www.icpsr.umich.edu/web/pages/NACJD/index.html.

## Ethics statement

The studies involving humans were approved by University of Oregon’s Institutional Review Board. The studies were conducted in accordance with the local legislation and institutional requirements. Written informed consent for participation in this study was provided by the participants’ legal guardians/next of kin.

## Author contributions

MS-C: Conceptualization, Data curation, Formal analysis, Funding acquisition, Investigation, Methodology, Project administration, Visualization, Writing – original draft, Writing – review & editing. CD: Conceptualization, Funding acquisition, Project administration, Visualization, Writing – original draft, Writing – review & editing. LL: Conceptualization, Funding acquisition, Writing – review & editing, Project administration.

## Footnotes

^1^The study was originally supported and funded through the Oregon Youth Authority and by Grant R01 DA015208 (P.I., Patricia Chamberlain, Ph.D.) from the National Institute on Drug Abuse, by Grant R01 MH054257 ^1cont.^ (P.I., Patricia Chamberlain, Ph.D.) from the National Institute of Mental Health, and by Grant R01 DA024672 (P.I., Leslie Leve, Ph.D.) from the National Institute on Drug Abuse.

^2^Given the richness of the longitudinal data in this study, we included multiple data points to characterize the experiences of the sample in adolescence and adulthood. Key variables were derived from the initial assessment wave and the most recent assessment wave (TPWS), along with variables that were best described by aggregation across all assessment waves (e.g., “AW” in Table 2).

## References

[ref1] Adler N. E. Boyce T. Chesney M. A. Cohen S. Folkman S. Kahn R. L.. (1994). Socioeconomic status and health: the challenge of the gradient. Am. Psychol. 49, 15–24. doi: 10.1037/0003-066X.49.1.15 8122813

[ref2] Aizer A. Doyle J. J. (2015). Juvenile incarceration, human capital, and future crime: evidence from randomly assigned judges *. Q. J. Econ. 130, 759–803. doi: 10.1093/qje/qjv003

[ref3] Anderson V. R. Walerych B. M. (2019). Contextualizing the nature of trauma in the juvenile justice trajectories of girls. J. Prev. Interv. Community 47, 138–153. doi: 10.1080/10852352.2019.1582141, PMID: 30849000

[ref4] Beck A. Harrison P. Guerino P. (2010). *Sexual victimization in juvenile facilities reported by youth, 2008–09.*U.S. Department of Justice, Bureau of Justice Statistics.

[ref5] Brennan T. Breitenbach M. Dieterich W. Salisbury E. J. Van Voorhis P. (2012). Women’s pathways to serious and habitual crime: a person-centered analysis incorporating gender responsive factors. Crim. Justice Behav. 39, 1481–1508. doi: 10.1177/0093854812456777

[ref6] Bureau of Justice Statistics. (2023). BJS releases preliminary statistics on incarcerated populations in 2022. *Bureau of Justice Statistics*. Available at: https://bjs.ojp.gov/document/pdrp22_pdrj22_pr.pdf

[ref7] Chamberlain P. Leve L. D. DeGarmo D. S. (2007). Multidimensional treatment foster care for girls in the juvenile justice system: 2-year follow-up of a randomized clinical trial. J. Consult. Clin. Psychol. 75, 187–193. doi: 10.1037/0022-006X.75.1.187, PMID: 17295579 PMC1995088

[ref8] Cioffi C. C. Schweer-Collins M. L. Leve L. D. (2022). Pregnancy and miscarriage predict suicide attempts but not substance use among dual-systems involved female adolescents. Child Youth Serv. Rev. 137:106494. doi: 10.1016/j.childyouth.2022.106494, PMID: 37089705 PMC10118061

[ref9] Cloitre M. Shevlin M. Brewin C. R. Bisson J. I. Roberts N. P. Maercker A.. (2018). The International Trauma Questionnaire: development of a self-report measure of ICD-11 PTSD and complex PTSD. Acta Psychiatr. Scand. 138, 536–546. doi: 10.1111/acps.12956, PMID: 30178492

[ref10] DeHart D. Lynch S. Belknap J. Dass-Brailsford P. Green B. (2014). Life history models of female offending: the roles of serious mental illness and trauma in women’s pathways to jail. Psychol. Women Q. 38, 138–151. doi: 10.1177/0361684313494357

[ref11] Derogatis L. R. Melisaratos N. (1983). The Brief Symptom Inventory: an introductory report. Psychol. Med. 13, 595–605. doi: 10.1017/S0033291700048017, PMID: 6622612

[ref12] Dierkhising C. B. Ko S. J. Woods-Jaeger B. Briggs E. C. Lee R. Pynoos R. S. (2013). Trauma histories among justice-involved youth: findings from the National Child Traumatic Stress Network. Eur. J. Psychotraumatol. 4:20274. doi: 10.3402/ejpt.v4i0.20274 PMC3714673 23869252

[ref9001] Dierkhising C. B. Eastman A. L. Chan K. (2023). Juvenile justice and child welfare dual system involvement among females with and without histories of commercial sexual exploitation. Children and Youth Services Review 150:106989.

[ref13] Dierkhising C. B. Lane A. Natsuaki M. N. (2014). Victims behind bars: a preliminary study of abuse during juvenile incarceration and post-release social and emotional functioning. Psychol. Public Policy Law 20, 181–190. doi: 10.1037/law0000002

[ref14] Eastman A. L. Putnam-Hornstein E. (2019). An examination of child protective service involvement among children born to mothers in foster care. Child Abuse Negl. 88, 317–325. doi: 10.1016/j.chiabu.2018.11.002, PMID: 30554123

[ref15] Eastman A. L. Schelbe L. McCroskey J. (2019). A content analysis of case records: two-generations of child protective services involvement. Child Youth Serv. Rev. 99, 308–318. doi: 10.1016/j.childyouth.2018.12.030

[ref16] Farrington D. P. (1986). Age and crime. (M. Tonry & Morris, N. Eds.; Vol. 7). Chicago, IL: University of Chicago Press.

[ref17] Felitti V. J. Anda R. F. Nordenberg D. Williamson D. F. Spitz A. M. Edwards V.. (1998). Relationship of childhood abuse and household dysfunction to many of the leading causes of death in adults. Am. J. Prev. Med. 14, 245–258. doi: 10.1016/S0749-3797(98)00017-8, PMID: 9635069

[ref18] Gottlieb A. Mahabir M. (2021). The effect of multiple types of intimate partner violence on maternal criminal justice involvement. J. Interpers. Violence 36, 6797–6820. doi: 10.1177/0886260518820705, PMID: 30600751

[ref19] Guidi J. Lucente M. Sonino N. Fava G. A. (2021). Allostatic load and its impact on health: a systematic review. Psychother. Psychosom. 90, 11–27. doi: 10.1159/000510696, PMID: 32799204

[ref20] Hammond I. Eastman A. Leventhal J. Putnam-Hornstein E. (2017). Maternal mental health disorders and reports to child protective services: a birth cohort study. Int. J. Environ. Res. Public Health 14:1320. doi: 10.3390/ijerph14111320, PMID: 29084185 PMC5707959

[ref21] Healy C. (2022). *Hawaii has no girls in juvenile detention. Here’s how it got there.* [The Washington Post]. Available at: https://www.washingtonpost.com/nation/2022/07/25/hawaii-zero-girls-youth-correctional-facility/

[ref22] Herz D. C. Dierkhising C. B. Raithel J. Schretzman M. Guiltinan S. Goerge R. M.. (2019). Dual system youth and their pathways: a comparison of incidence, characteristics and system experiences using linked administrative data. J. Youth Adolesc. 48, 2432–2450. doi: 10.1007/s10964-019-01090-3, PMID: 31385232

[ref23] Herz D. C. Eastman A. L. Putnam-Hornstein E. McCroskey J. (2021). Dual system youth and their pathways in Los Angeles County: a replication of the OJJDP Dual System Youth Study. Child Abuse Negl. 118:105160. doi: 10.1016/j.chiabu.2021.105160, PMID: 34175505

[ref24] Holman B. Ziedenberg J. (2013). *The danger of detention: the impact of incarcerating youth in detention and other secure facilities*. Justice Policy Institute. Available at: https://justicepolicy.org/wp-content/uploads/2022/02/06-11_rep_dangersofdetention_jj.pdf

[ref25] Johansson P. Kempf-Leonard K. (2009). A gender-specific pathway to serious, violent, and chronic offending: exploring Howell’s risk factors for serious delinquency. Crime Delinq. 55, 216–240. doi: 10.1177/0011128708330652

[ref26] Keenan K. Hipwell A. Chung T. Stepp S. Stouthamer-Loeber M. Loeber R.. (2010). The Pittsburgh Girls Study: overview and initial findings. J. Clin. Child Adolesc. Psychol. 39, 506–521. doi: 10.1080/15374416.2010.486320 20589562 PMC2946599

[ref27] Kerig P. K. (2018). Polyvictimization and girls’ involvement in the juvenile justice system: investigating gender-differentiated patterns of risk, recidivism, and resilience. J. Interpers. Violence 33, 789–809. doi: 10.1177/0886260517744843, PMID: 29411692

[ref28] Kerig P. K. Becker S. P. (2010) in From internalizing to externalizing: theoretical models of the processes linking PTSD to juvenile delinquency. ed. Egan S. (Hauppauge, NY: Nova Science Publishers)

[ref29] Kerig P. Schindler S. (2013). Engendering the evidence base: a critical review of the conceptual and empirical foundations of gender-responsive interventions for girls’ delinquency. Laws 2, 244–282. doi: 10.3390/laws2030244

[ref30] Kerr D. C. R. Leve L. D. Chamberlain P. (2009). Pregnancy rates among juvenile justice girls in two randomized controlled trials of multidimensional treatment foster care. J. Consult. Clin. Psychol. 77, 588–593. doi: 10.1037/a0015289 19485598 PMC2706574

[ref31] Kewley S. (2017). Strength based approaches and protective factors from a criminological perspective. Aggress. Violent Behav. 32, 11–18. ISSN 1359-1789. doi: 10.1016/j.avb.2016.11.010

[ref32] Lehavot K. Katon J. G. Chen J. A. Fortney J. C. Simpson T. L. (2018). Post-traumatic stress disorder by gender and veteran status. Am. J. Prev. Med. 54, e1–e9. doi: 10.1016/j.amepre.2017.09.008, PMID: 29254558 PMC7217324

[ref33] Leve L. D. Khurana A. Reich E. B. (2015). Intergenerational transmission of maltreatment: a multilevel examination. Dev. Psychopathol. 27, 1429–1442. doi: 10.1017/S0954579415000851, PMID: 26535935 PMC4635523

[ref34] Leve L. D. Schweer-Collins M. Bates E. (2022). Criminal offense charges in women: a 10-year follow-up of an RCT of treatment foster care Oregon. J. Consult. Clin. Psychol. 90, 901–910. doi: 10.1037/ccp0000764, PMID: 36326664 PMC9892281

[ref35] Lynch S. M. DeHart D. D. Belknap J. Green B. L. (2012). *Women’s pathways to jail: the roles & intersections of serious mental illness & trauma: (528222013–001)* [dataset]. Available at: 10.1037/e528222013-001

[ref36] McCarthy P. Schiraldi V. Shark M. (2016). The future of youth justice: a community-based alternative to the youth prison model. New Thinking in Community Corrections Bulletin. Washington, D.C.: U.S. Department of Justice, National Institute of Justice, NCJ 250142

[ref37] McEwen B. S. Stellar E. (1993). Stress and the individual: mechanisms leading to disease. Arch. Intern. Med. 153:2093. doi: 10.1001/archinte.1993.00410180039004 8379800

[ref38] Mendel R. (2022a). Diversion: a hidden key to comabting racial and ethnic disparitiesin Juvenile Justice. The Sentencing Project. Available at: http://www.sentencingproject.org/reports/diversion-a-hidden-key-to-combating-racial-and-ethnic-disparities-in-juvenile-justice/ (Accessed November 2, 2022).

[ref39] Mendel R. (2022b). *Why youth incarceration fails: an updated review of the Evidence*. The Sentencing Project. Available at: https://www.sentencingproject.org/app/uploads/2023/03/Why-Youth-Incarceration-Fails.pdf

[ref40] Mendel R. (2023). *Effective alternatives to youth incarceration.* The Sentencing Project. Available at: https://www.sentencingproject.org/reports/effective-alternatives-to-youth-incarceration/

[ref41] Moffitt T. E. (1993). Adolescence-limited and life-course-persistent antisocial behavior: a developmental taxonomy. Psychol. Rev. 100, 674–701. doi: 10.1037/0033-295X.100.4.674 8255953

[ref42] Mulvey E. P. (2004). Introduction: pathways to Desistance Study. Youth Violence Juvenile Justice 2, 211–212. doi: 10.1177/1541204004266287 PMC2813466 20119515

[ref43] Obidoa C. A. Reisine S. L. Cherniack M. (2010). How does the SF-36 perform in healthy populations? A structured review of longitudinal studies. J. Soc. Behav. Health Sci. 4, 30–48. doi: 10.5590/JSBHS.2010.04.1.02

[ref44] Office of the Assistant Secretary for Planning and Evaluation [ASPE]. (2023). *HHS Poverty Guidelines for 2023.* U.S. Health and Human Services. Available at: https://aspe.hhs.gov/topics/poverty-economic-mobility/poverty-guidelines

[ref45] Piquero A. R. Farrington D. P. Blumstein A. (2003). The criminal career paradigm, vol. 30. Chicago, IL: University of Chicago Press, 359–506

[ref46] Putnam-Hornstein E. Cederbaum J. A. King B. Eastman A. L. Trickett P. K. (2015). A population-level and longitudinal study of adolescent mothers and intergenerational maltreatment. Am. J. Epidemiol. 181, 496–503. doi: 10.1093/aje/kwu321, PMID: 25740788

[ref47] Putnam-Hornstein E. Hammond I. Eastman A. L. McCroskey J. Webster D. (2016). Extended foster care for transition-age youth: an opportunity for pregnancy prevention and parenting support. J. Adolesc. Health 58, 485–487. doi: 10.1016/j.jadohealth.2015.11.015, PMID: 26853490

[ref48] Puzzanchera C. (2021). *Juvenile arrests, 2019*. Office of Juvenile Justice and Delinquency Prevention. Available at: https://ojjdp.ojp.gov/library/publications/juvenile-arrests-2019

[ref49] Puzzanchera C. Sladky T. J. Kang W. (2023). *Easy access to the census of juveniles in residential placement: 1997-2021.* Office of Juvenile Justice and Delinquency Prevention. Available at: https://www.ojjdp.gov/ojstatbb/ezacjrp/

[ref50] Reilly J. J. Kelly J. (2011). Long-term impact of overweight and obesity in childhood and adolescence on morbidity and premature mortality in adulthood: systematic review. Int. J. Obes. 35, 891–898. doi: 10.1038/ijo.2010.222, PMID: 20975725

[ref51] Rhoades K. A. Leve L. D. Harold G. T. Kim H. K. Chamberlain P. (2014). Drug use trajectories after a randomized controlled trial of MTFC: associations with partner drug use. J. Res. Adolesc. 24, 40–54. doi: 10.1111/jora.12077, PMID: 24729667 PMC3979629

[ref52] Rodermond E. Kruttschnitt C. Slotboom A.-M. Bijleveld C. C. (2016). Female desistance: a review of the literature. Eur. J. Criminol. 13, 3–28. doi: 10.1177/1477370815597251

[ref53] Ronel N. Elisha E. (2011). A different perspective: introducing positive criminology. Int. J. Offender Ther. Comp. Criminol. 55, 305–325. doi: 10.1177/0306624X09357772, PMID: 20103584

[ref54] Ronel N. Segev D. (2014). Positive criminology in practice. Int. J. Offender Ther. Comp. Criminol. 58, 1389–1407. doi: 10.1177/0306624X13491933 23782705

[ref55] Saar M. Epstein R. Rosenthal L. Vafa Y. (2015). *The sexual abuse to prison pipeline: the girls’ story.* Available at: http://forwomen.org/%5Cfiles/documents%5CSexual%20Abuse%20to%20Prison%20Pipeline%20The%20Girls%20Story%202015.pdf http://hdl.handle.net/11212/2317

[ref56] Sobell L. C. Sobell M. B. (1992). “Timeline follow-back” in Measuring alcohol consumption. eds. Litten R. Z. Allen J. P. (Totowa, NJ: Humana Press), 41–72.

[ref57] Spanier G. B. (1976). Measuring dyadic adjustment: new scales for assessing the quality of marriage and similar dyads. J. Marriage Fam. 38:15. doi: 10.2307/350547

[ref58] Spivak A. L. Wagner B. M. Whitmer J. M. Charish C. L. (2014). Gender and status offending: judicial paternalism in juvenile justice processing. Fem. Criminol. 9, 224–248. doi: 10.1177/1557085114531318

[ref59] Straus M. A. Douglas E. M. (2004). A Short Form of the Revised Conflict Tactics Scales, and typologies for severity and mutuality. Violence Vict. 19, 507–520. doi: 10.1891/vivi.19.5.507.63686 15844722

[ref9002] Swedo E. A. Aslam M. V. Dahlberg L. L. (2023). Prevalence of Adverse Childhood Experiences Among U.S. Adults — Behavioral Risk Factor Surveillance System, 2011–2020. MMWR. Morb. Mortal. Wkly. Rep. 72, 707–715. doi: 10.15585/mmwr.mm7226a2 37384554 PMC10328489

[ref60] Teplin L. A. Abram K. M. Washburn J. J. Welty L. J. Hershfield J. A. Dulcan M. K. (2013). *The northwestern juvenile project: overview* Office of Juvenile Justice and Delinquency Prevention.

[ref61] Vera Institute. (2023). *Vera takes on girls’ incarceration in the country’s most populous state*. Available at: https://www.vera.org/ending-mass-incarceration/reducing-incarceration/reducing-jail-and-prison-population/ending-girls-incarceration-initiative/egi-ca-action-network#:~:text=In%20April%202023%2C%20Vera%20and,Vera%20to%20end%20girls’%20incarceration

[ref62] Ware J. E. Sherbourne C. D. (1992). The MOS 36-Item Short-Form Health Survey (SF-36): I. conceptual framework and item selection. Med. Care 30, 473–483. doi: 10.1097/00005650-199206000-00002 1593914

[ref63] World Health Organization [WHO]. (2021). *Hypertension*. Available at: https://www.who.int/westernpacific/health-topics/hypertension

[ref64] Xu H. Cupples L. A. Stokes A. Liu C.-T. (2018). Association of obesity with mortality with over 24 years of weight history: findings from the Framingham Heart Study. JAMA Netw. Open 1:e184587. doi: 10.1001/jamanetworkopen.2018.4587, PMID: 30646366 PMC6324399

[ref65] Zahn M. A. Agnew R. Fishbein D. Miller S. Dakoff G. Kruttschnitt C.. (2010). Causes and correlates of girls’ delinquency. Office of Juvenile Justice and Delinquency Prevention.

