# Supplementary material for: The long-term collateral consequences of juvenile justice involvement for females
Source: Front Psychol. 2024 Jan 8;14:1321355. doi: 10.3389/fpsyg.2023.1321355 (PMC10800427; doi:10.3389/fpsyg.2023.1321355)
Supplement: Supplementary file 1 [file Data_Sheet_1.docx]

SUPPLEMENTAL FILE A

CHARACTERISTICS OF LIVING SITUATION INTERVIEW

***A. PAST LIVING SITUATIONS: FROM LAST INTERVIEW TO PRESENT***

***We’d like to take a quick “snapshot” of every place you have lived since the last time we talked to you. This could include your own home or apartment, friend’s houses, relative’s houses, homeless shelters, correctional facilities, hospitalizations, treatment programs, military, and even just crashing at people’s houses. And for each snapshot, I will ask you the city and state, the dates (as best as you can remember) and why you left.***

**01a. What kind of setting or place were you living in / staying in?** **And why did you leave?**

Record each change of setting (place).

**01b. What city and state is that in**? ______________ ____

**What were the dates you lived there?**

**01c. From** __ __ / __ __ / __ __

**01d. To** __ __ / __ __ / __ __

**01e. Why did you leave?** (__ __) __________________

[For each additional setting]: And then where were you living/staying ?

*****Repeat for all additional living situations 02 – 10. If more than 10 living situations, record on ‘extra’ pages.*****

**######################################################################################**

***Code each setting carefully.***

***If unclear which code to use, write your suggestions and rationale on a post-it on the instrument and send an email re: issue to assess coordinator.***

***In any situation where you have had to record something unusual or have made some kind of decision that isn’t a straightforward coding, write a note to data management about what you have done regarding dates, placements, etc so they can add it to the list of atypical situations and make sure it is in alignment with the rest of these types of situations.***

***Characteristics of Living Situations (CLS) Placement Codes***

**01 Living by yourself / living with peers or non-relatives (if TC is adult)**

Could be a home, or apt., or trailer or other house-like dwelling

a. with peers or partner,

b. with relatives, including bioparents living in TC’s dwelling

c. with relatives, shared housing which is not the bioparent’s housing

d. in own apt even if it is part of a halfway house program; will differentiate between

living in own apt/house (01) and living in a corrections group halfway house (10)

and living in a substance tx group halfway house (11)

*Not code 01*: *TC living with bioparents in bioparents’ dwelling is coded 03*

**02 In-patient residential treatment for psychiatric hospital care**

a. psychiatric hospital care, any length of time, even 1 day

*Not code 02*:

-*Substance related hospital stays, such as detox in a hospital setting, are coded as 17*

*-Hospitalization for surgery, pregnancy, etc (not psychiatric, not substance) is coded 17*

**03 Biological parent’s home**

a. TC stays at her parent’ s dwelling (regardless of paying rent or not)

*Not code 03*:

-*bioparents staying in TC’s dwelling is coded 01*

*-bioparents and TC sharing a dwelling, not primarily associated with one or the other of them, is coded 01*

**04 Relative’s home**

a. TC stays at her relative’ s dwelling (regardless of paying rent or not)

*Not code 04*: *-relatives and TC sharing a dwelling, not primarily associated with one or the other of them, is coded 01*

**05 Non-relative adult’s home (CHOOSE THIS OPTION FOR MINORS ONLY)**

a. frequently this is TC staying with an older friend, an unrelated adult like a boyfriend’s mother

*Not code 05*: *-TC 18+*

**06 Camper/tent** **on someone’s property, with their permission**

a. TC stays in a camper or tent in someone’s yard, street, driveway etc, (regardless of paying rent or not)

b. not relevant who the camper / tent belongs to

*Not code 06*: *-if TC is squatting somewhere with a tent or camper, moving around, code as 12*

**07 College dorm / sorority house / Job Corps dorm**

a. TC stays in a dorm or dorm-like setting with multiple beds / rooms under some sort of program or supervision

*Not code 07*: *-group home, treatment center, halfway house code as 10*

*-substance use treatment program, home or setting, code as 11*

***Characteristics of Living Situations (CLS) Codes con’t.***

**08 Foster home = foster family**

a. This code is for a foster family setting

*Not code 08*: *-foster group home code as 10*

**THESE must be coded on a “one day” basis. Capture every day, even one day, that TC spent in any of these “critically important to the research” settings.**

***09 Jail/Prison/Detention/Correctional Facility**

a. If TC is moving from one facility to another, within the prison system, code as one setting, until she arrives in the next setting. Do not create a separate setting for the transit to the setting, even if more than one day. She is still in the care of that first prison setting until she arrives in the next prison setting.

***10 Group home, residential treatment center (not substance) / home, group halfway house** (for corrections)

a. This code is for a group home setting. A foster family home with a lot of people in it, is not by size alone, a group home. Be sure to probe.

b. Apply this code to other group residential settings that have some kind of specific intent and/or affiliation related to delinquency or incarceration and monitoring or supervision and in which a group of people live together in a facility or dwelling

*Not code 10*: *-foster family home code as 08*

*-TC living in her own apt or dwelling in an apt complex that is a halfway house for corrections; code this situation of living in own apt as 01*

***11 Substance use treatment program or home, or group halfway house for substance use**

a. Apply this code to other residential settings that have some kind of specific intent of and/or affiliation related to substance use tx of follow-up to treatment and some level of monitoring or supervision.

*Not code 11*: *-TC living in her own apt or dwelling in an apt complex that is a halfway house for substance use; code this situation of living in own apt as 01*

*-not a hospital visit for detox or overdose; code those as 17*

***12 Homeless**

a. This code is for living on the streets, or living in a car = not having a dwelling to live or sleep in

b. This code applies in a situation where TC is in her car for a period of time, looking for a place to live or stay, not having anywhere to live or stay

*Not code12*: *-homeless shelter or temporary shelter is code 13*

*-TC is on vacation or planned trip and is sleeping in her car = not a setting change*

*-TC is moving to a new location and sleeping in her car or riding on a bus*

*along the way = record as prior setting until arrival at new setting*

*-TC is on a bus traveling somewhere or is hitchhiking or hopping trains to a destination*

***13 Homeless shelter / temporary shelter**

a. This code is for staying in any kind of homeless shelter, or any facility like a church or gym that is being used as a homeless shelter or temporary shelter

b. It is very important that this is captured and differentiated from “homeless”. It can be a within a period of being homeless, living on the streets, in a car or squatting which is code 12. If during this period of homelessness, there was even one night in a shelter, you need to capture that time with a code 13.

**15 Military living situation**

a. This code is for housing provided by military during times of training or service that are not a typical living situation like a house or apt..This code is for something like living in a barracks or on a Navy ship, or in a war or other comparable situation.

*Not code15*: *-living on own, in apt, on a base or in military housing while working in the military; code as 01*

**16 Other:** (specify: ______________________)

**17 General hospitalizations**

a. This code is for general hospitalizations, stays in hospitals

*Examples would be for a birth, a surgery, suicide attempt, detox, overdose etc*

*Not code17*: *-hospitalization in psychiatric facility or ward, stay for psychiatric reasons is code 02*

*-if hospitalized for an accident or surgery and then moved to a residential physical rehab center, code rehab portion as 16 OTHER with an explanation*

**14 Random living situations or “crashing”** (in any settings listed in code 01-08 and 17)

**Random living situations for short periods at friends or relatives or other places not coded as 09 – 15, in situations when TC cannot identify the time frame and can’t sort out the settings but it is less than 14 days. If setting is 14 days or more, do not code as 14, code** **with 01-08.**

**Use this code in the following situations of Codes 01-08 and 17:**

1. TC is staying in places coded 01 -08 or 17 for less than two weeks at a time.

2. TC is moving from place to place coded 01-08 or 17 with such frequency that TC cannot remember dates and you cannot make a setting by setting record of where she was living = very frequent changes and settings which TC stays in for 13 days or less**.**

**If TC is in settings 09 – 15, you must make a placement setting for these, even if it is just one day.**

**Example 1:**

14 TC goes between 4 different places, a different place every night for 30 days. She doesn’t stay longer than one night at any place. She isn’t in any of the 09-15 settings during this period.

**Example 2:**

12 TC can’t remember where she stayed for most of May, mostly on the streets or in cars

13 but she remembers that she stayed in a women’s homeless shelter for a weekend and

14 then she stayed with people her friend knows for a couple of nights and some other peoples’ house for another few nights, and then these other people for about a week – can’t remember the dates but total time was about 3 weeks and she didn’t stay anywhere for more than 13 days, just back and forth in these different people’s houses (not 09-15 places like jail or tx or group home or shelter)

09 then went to jail for a week and

12 then was living on the streets again and then

06 then stayed with some other people in their tent in someone’s backyard at their house and

12 then they gave her the tent which she set up by the river with some other people and

04 then stayed at her uncle’s house for a few days and

14 then went to three different places where she just slept, doesn’t know the people and doesn’t know the dates of each setting, and no setting was more than 13 days – just have to group them together as “random living situations”

**NOTES:**

**-Do not include on CLS:** vacations/travels, transitions

**-If TC vacillates between crashing and homelessness, or between homelessness and homeless shelters**: try to get an approximation of how many days she was in the street vs in shelters or crashing. Divide the total period into two placements and apply the appropriate number of days to each setting.

Example 1:

For 2 months, TC was back and forth between homeless and in a homeless shelter every few days. She could only stay in the shelter 2 nights in a row, so she did that every few days. She estimates that she went to the homeless shelter about 10 times and stayed two nights each time. Take the entire time period, and assign 20 days to a homeless shelter(13) and the remaining 40 days to homeless (12). When you do this, write a note to data management about what you did.

**-Very important to capture all days in jail, homeless, or in TX, no matter how short the period, no matter if only one day.**

**-The CLS is NOT designed to capture economic dependence**. Placement category 01 is a broad category that captures any instance in which adult TCs are living with non-relatives (whether dependent or not), as well as any situation where a TC (adult or minor) is living on her own or with peers. It is also used for instances of TC opening her home to her relatives, parents, etc. and sharing a home (does not belong to either of them or belongs equally to each of them)
