# Supplementary material for: The long-term collateral consequences of juvenile justice involvement for females
Source: Front Psychol. 2024 Jan 8;14:1321355. doi: 10.3389/fpsyg.2023.1321355 (PMC10800427; doi:10.3389/fpsyg.2023.1321355)
Supplement: Supplementary file 2 [file Data_Sheet_2.docx]

SUPPLEMENTAL FILE B

ADVERSE CHILDHOOD EXPERINCES REVISED COMPOSITE MEASURE

| **Table S1** | | | |
| --- | --- | --- | --- |
| *Adverse Childhood Experiences Revised Composite* | | | |
| **ACE Item** | **(Form or Instrument) Question(s)** | **Respondent** | **Coding** |
| 1. Child Emotional Abuse | Official Child Welfare Report of Child Emotional Abuse | Official Child Welfare Reports | 0 = no, 1 = yes |
| 2. Child Physical Abuse | (Referral Form) Is there any documented physical abuse of TC? | Caseworker Report | 0 = no, 1 = yes |
| 3. Child sexual abuse | (Referral Form) Is there any documented sexual abuse of TC? | Caseworker Report | 0 = no, 1 = yes |
| 4. Emotional Neglect | (Assessing Environments; Berger et al., 1988)  95. I never felt that my parents really loved me.  138. I felt rejected by my parents. | Participant | 0 = no, 1 = yes (if answered yes on any one of the two questions) |
| 5. Child Neglect | Official Child Welfare Report of Child Neglect | Official Child Welfare Reports | 0 = no, 1 = yes |
| 6. Parent / Caregiver Divorce | (Referral Form) Parents divorced during this child’s lifetime | Caseworker Report | 0 = no, 1 = yes |
| 7. Interpersonal Family Violence | (Referral Form) Family violence -- weapons used or arrested for or victim of (e.g., murder, shot); exclude sexual abuse | Caseworker Report | 0 = no, 1 = yes |
| 8. Parent / Caregiver Substance Use Problem | (Referral Form) Does this youth's bio mom/dad/step/adopted parents have a history of drug or alcohol abuse? | Caseworker Report | 0 = no, 1 = yes (if answered yes to any one of the three included questions) |
| 9. Parent / Caregiver Mental Illness | (Referral Form) Bio/Adopted Dad/Mom hospitalized for mental illness | Caseworker Report | 0 = no, 1 = yes (if answered yes to any one of the two included questions) |
| 10. Parent / Caregiver Incarceration | (Referral Form) Have any of this youth's mom/dad/step/adopted parents ever been convicted of a crime? | Caseworker Report | 0 = no, 1 = yes (if answered yes to any one of the three included questions) |
